# Phenol Abatement by Titanium Dioxide Photocatalysts: Effect of The Graphene Oxide Loading

**DOI:** 10.3390/nano9070947

**Published:** 2019-06-29

**Authors:** Usuma Naknikham, Giuliana Magnacca, Ang Qiao, Peter Kjær Kristensen, Vittorio Boffa, Yuanzheng Yue

**Affiliations:** 1 Department of Chemistry and Bioscience, Aalborg University, 9220 Aalborg, Denmark; 2 Dipartimento di Chimica, Universitá di Torino, 10125 Torino, Italy; 3 State Key Laboratory of Silicate Materials for Architectures, Wuhan University of Technology, Wuhan 430070, China; 4 Department of Physics and Nanotechnology, Aalborg University, 9220 Aalborg, Denmark

**Keywords:** photocatalytic activity, water purification, TiO_2_, Graphene, reproducibility

## Abstract

Hetero-photocatalytic graphene-TiO_2_ materials have, in the literature, been found to possess better photocatalytic activity for environmental applications compared to pure TiO_2_. These types of materials can be prepared in different ways; however, their photocatalytic performance and quality are not easily controlled and reproduced. Therefore, we synthetized graphene oxide-TiO_2_ nanoparticles by sol-gel reaction from TiCl_4_, as precursor, with two different methods of synthesis and with a graphene oxide (GO) loading ranging from 0 to 1.0. This approach led to a good adhesion of GO to TiO_2_ through the Ti-O-C bonding, which could enhance the photocatalytic performances of the materials. Overall, 0.05 wt % GO loading gave the highest rate in the photodegradation of phenol under visible light, while higher GO loadings had a negative impact on the photocatalytic performances of the composites. The 0.05 wt % GO-TiO_2_ composite material was confirmed to be a promising photocatalyst for water pollutant abatement. The designed synthetic approach could easily be implemented in large-scale production of the GO-TiO_2_ coupling materials.

## 1. Introduction

More than 80% of wastewater around the world is today released to the environment without appropriate treatment [1]. Phenolic compounds are among the possible pollutants that impact natural aquatic systems. They are used in agriculture and are present in many industrial processes, such as pulp and paper, synthesis of pharmaceuticals, oil refining, production of polymers and resins, and food processing [2,3,4,5]. As a result, phenols are now often found in wastewater and in natural water. The US Environmental Protection Agency (USEPA), the National Pollutant Release Inventory (NPRI) of Canada and the European Union (EU) consider phenols and their derivatives as priority pollutants, due to their serious impact on humans, animals and the aquatic environment [2,4]. Therefore, wastewater treatment plants are requested to decrease the concentration of those compounds to the safety level, namely below 0.1–1.0 ppm [3,4]. Since phenols and phenolic compounds are chemically stable and highly soluble in water [3], phenols abatement is a challenge for the traditional wastewater treatment plants. In addition, advance tertiary wastewater treatment technologies are still costly and often require consumption of additional chemicals and energy [2,3].

In this context, solar photocatalysis has the potential to offer feasible solutions for the abatement of phenols and other emerging pollutants [6]. Titanium dioxide (TiO_2_) is the most common material for the photocatalytic depollution of water, because it is chemically stable, nontoxic, relatively inexpensive, and it shows high degradation activity under UV light [6,7,8]. However, the main drawbacks of TiO_2_ photocatalysts are wide band bap energy (3.2 eV), which allows exploiting only about 5% of sunlight, and the fast recombination of electron-hole (e^−^-h^+^) pairs [3,6]. Recently, the use of graphene in combination with semiconductor materials, such as TiO_2_ [5,6], has shown large potential for the photocatalytic depollution of both water and air. Indeed, the combination of graphene-based structure with TiO_2_ can narrow the band gap energy and decrease of the rate of e^−^-h^+^ pair recombination [6,9], resulting in a wide light absorption range: from UV to visible light. Therefore, the synergy of GO with TiO_2_ and enhanced efficiency in the photodegradation of organic pollutants can be achieved by using the GO-TiO_2_ composites.

As reported in our previous work [10] and by other authors [6,9,11], in-situ nucleation and growth of TiO_2_ nanoparticles on GO sheets allows achieving strong Ti-O-C chemical linkage between the two materials, and thus enhances photocatalytic activity. The synthesis conditions have a strong impact on the structure of the photocatalysts, and therefore on their ability to degrade water pollutants [12]. For instance, the pH of the reaction mixture can allow a strong electrostatic interaction between TiO_2_ and graphene oxide [3,10,13]. In addition, graphene oxide is partially reduced during synthesis, due to the reaction temperature and to the interaction of its functional groups with the surface of TiO_2_ particles [5,9,14,15,16], thus photocatalytic activity is improved by the surface bonding, which facilitates electron transfer from TiO_2_ to the graphene-based electron acceptor [17].

The photodegradation activity is not only affected by the interface bonding between GO sheets and TiO_2_ nanoparticles, but also by the GO loading. Indeed, a higher amount of GO can lower the photodegradation rate, because the excess of GO can prevent light to reach the TiO_2_ photocatalytic centers [17,18,19]. Conversely, enough GO should be added to establish a wide interface with TiO_2_ nanoparticles [20]. In this work, the TiO_2_ photocatalysts was loaded with GO in different weight ratios (0–1.0 wt %) to study the impact of different GO loading over the TiO_2_ photocatalyst. The structural features and the photocatalytic activity of the new materials were investigated. Over the past years, GO-TiO_2_ photocatalysts have been mostly tested by degradation of organic dyes. However, most of the emerging organic pollutants are often less prone to mineralization by photocatalysis than the common organic dyes, having a different electron structure and a less extended conjugation [21,22,23]. Hence, in this study, we used phenol as model pollutant and we performed photocatalytic tests under simulated sunlight, as this is the most convenient way to use photocatalytic oxidation in real wastewater plants. Furthermore, since there are concerns about synthesis reproducibility when different reactors are used, two fabrication methods were used: (a) the reaction mixture was stirred in a closed Pyrex beaker; and (b) GO-TiO_2_ suspension was kept in a static Teflon vessel. Thus, it was possible to compare the morphology and the photocatalytic performances of the materials prepared under stirring and in static conditions.

## 2. Experimental Section

After preparing graphene oxide (GO) via a modified Hummers method from a natural graphite powder (UF2 99,5 Graphit Kropfmühl GmbH, Hauzenberg, Germany) as described elsewhere, [10], the graphene-titanium dioxide (GT) composites were synthesized by two methods, namely in static conditions (GTS) and in a stirred tank (GTD), and their properties were compared. All chemicals used for the synthesis of the nanocomposites were purchased from Sigma-Aldrich (St. Louis, MO, USA), unless otherwise specified.

### 2.1. Synthesis of the GT Composites

The ratios of TiCl_4_/GO/H_2_O in the synthetic mixtures were calculated to obtain a final GO + TiO_2_ concentration of 1.0 g/L. In short, a freeze-dried GO powder was dispersed at 0.01 wt % in ultrapure water (resistivity ≥ 18 MΩ cm) by high power ultrasonication in a cold bath for 3 h. The concentration of the GO suspension was adjusted with ultrapure water according to the above calculation to total volume of 100 mL. The mixture was sonicated for 30 min and then stirred in an ice bath for 30 min. TiCl_4_ (98% purity) was added under vigorous stirring for 1 h in the ice bath. After allowing the mixture to reach room temperature, the pH was adjusted to 6.0 with NH_4_OH (25% in water), and maintained at this value for 2 h. For the static (S) process, the mixture was transferred to Teflon-lined autoclave, maintained at 100 °C for 4 h in an oven and let cool overnight. In the dynamic (D) method, the suspension was heated at 100 °C for 4 h and cooled down to room temperature with continuous stirring for 18 h. After this, GT nanocomposites were collected by centrifugation and cleaned with ultrapure water until no chloride ions were detected by the silver nitrate test (0.1 M AgNO_3_ solution) on supernatant. Finally, the precipitation was washed with ethanol and dried in vacuum at 40 °C.

GT photocatalysts with theoretical GO loading of 0.05, 0.1, 0.2, 0.5 and 1.0 wt %, named 0.05GTS, 0.1GTS, 0.2GTS, 0.5GTS and 1.0GTS, respectively, were prepared by the static process Samples 0.05GTD and 1.0GTD (0.05 and 1.0 wt % GO loading, respectively) were synthesized with under stirring conditions. A pure TiO_2_ reference, TS, was prepared by the statistic method, in the same conditions used for the GTS composites with no GO in the starting mixture.

### 2.2. Characterization of the GT Composites

The morphology of the samples was characterized by high-resolution transmission electron microscopy (HRTEM) over a JEOL 3010-UHR instrument (Tokyo, Japan). The crystalline phase and the TiO_2_ crystallites size were investigated by a PANAnalytical Empyrean diffractometer (Almelo, The Netherlands), operating at 45 kV and 40 mA, with Cu Kα radiation (λ = 1.5418 Å). Both reflection spinner and SAXS (small angle X-ray scattering) stage were used. SAXS measurements were performed over 5.3 × 10^−3^ Å^−1^ and 3.5 × 10^−1^ Å^−1^ for studying the size distribution of the titania particles. The X-rays diffraction (XRD) patterns of the powdered samples were acquired in a 2*θ* range from 5° to 70°. Reference cards, PDF 01-021-1272 of anatase and 01-029-1360 of brookite, were chosen for peak analysis [24]. The fraction of the anatase phase, $W_{A}$, over the total crystalline material (anatase and brookite) was calculated by Equation (1) [25,26].

(1) $$W_{A}=\frac{k_{A}A_{A}}{k_{A}A_{A}+k_{B}A_{B}}$$

where $A_{A}$ is the integrated intensity of anatase phase highest peak (101), $A_{B}$ is the integrated intensity of brookite phase highest peak (121), and the coefficients $k_{A}$ and $K_{B}$ are 0.886 and 2.721, respectively [10]. The deconvolution technique was used for anatase and brookite peak separation due to their overlapping. After baseline subtraction, the XRD pattern was fitted by Lorentzian function over the Fityk 0.9.8 (freeware software developed by Marcin Wojdyr). By doing that, it was assumed that the broadening of the three main peaks of brookite is the same [24].

XPS spectra were obtained by an ESCALAB 250Xi X-ray photoelectron spectrometer (Thermo Fisher Scientific, Waltham, MA, USA) using Al Kα radiation. The diffuse reflectance spectra in the range 200–800 nm were measured by UV-visible Spectrophotometer, PerkinElmer Lambda 1050 (Waltham, MA, USA), with an integrating sphere using BaSO_4_ as a reference material. The band gap energy was obtained from the Tauc plot of the Kubelka-Munk (K-M) function [27,28,29]:

(2) $${\left(\alpha hv\right)}^{\frac{1}{2}}=C\left(hv-E_{g}\right)$$

where α is the absorption coefficient of the solid at a certain value of wavelength (λ), *h* is Planck’s constant, C is the proportionality constant, v is the frequency of light and $E_{g}$ is the band-gap energy.

### 2.3. Photocatalytic Tests

**Figure 1:**
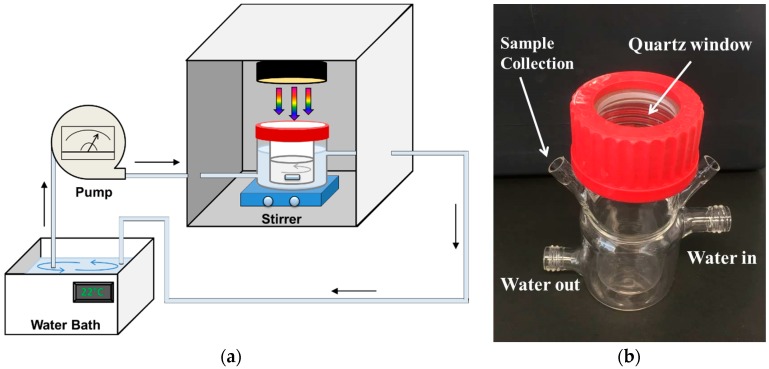
(**a**) Schematic of the set-up for photocatalytic tests; and (**b**) photocatalytic cell.

Phenol was used as the model pollutant for studying the photodegradation activity of the GT composites under sun simulator. The set-up of photocatalytic experiment is shown in Figure 1a: the photocatalytic double wall cell was made of borosilicate glass (capacity 30 mL) with a quartz window cap (Figure 1b, diameter 30 mm) to exploit the simulated sunlight. A control cell with the same dimensions was used for tests in the dark and therefore was covered with a polypropylene blind cap. The solar light was generated at 1000 W/m^2^ intensity by a 300 W xenon lamp (model LS0306, LOT QuantumDesign, San Diego, CA, USA) and the calibration was done before testing with a Si reference solar cell (Model LS0042, ReRa Solutions, Nijmegen, The Netherlands). The lamp was set up above the photocatalytic cell at a distance of 100 mm from the surface of the sample. The temperature for the photodegradation experiment was controlled by water circulation at 22 ± 1 °C.

The GT composites were dispersed in ultrapure water at concentration 1000 ppm with ultrasonication until well dispersed. After that, the sample suspension and 20 ppm phenol solution with ratio 1:1 were mixed in the photocatalyst cells without light for 30 min, because we observed that after this time adsorption-desorption equilibrium was reached. Then, the test suspension was exposed to the simulated solar light and the samples were collected at the specific time intervals throughout 180 min. The collected samples were filtrated through 0.45 μm cellulose filter. The sample solutions were analyzed via High Performance Liquid Chromatography (HPLC, Dionex with Chromeleon 6.80 software (Thermo Fisher Scientific, Waltham, MA, USA) with a Luna^®^ 5μ C18(2) 100Å column (Phenomenex, Torrance, CA, USA), 250 × 4.60 mm^2^. KH_2_PO_4_ (0.025 M) solution and acetonitrile with ratio 40:60 were used as mobile phase.

The apparent rate constant for the photodegradation tests (k) [29,30] was obtained from the plot of −($ln\frac{C}{C_{0}}$) against with time (minutes), i.e., according to a first-order kinetic:

(3) $$-\ln\left(\frac{C}{C_{0}}\right)=kt$$

where k is the apparent kinetic constant of pseudo-first order, $C_{0}$ is the starting concentration and C is the concentration at the reaction time (t).

## 3. Results and Discussion

### 3.1. Morphology of GT Nanocomposites

In this work, we present and discuss the effects of GO loading (from 0–1.0 wt %) on the photocatalytic activity of GT composites. Moreover, some of these materials were synthesized both under stirring and in static conditions and their differences in terms of morphology and photocatalytic activity were investigated.

**Figure 2:**
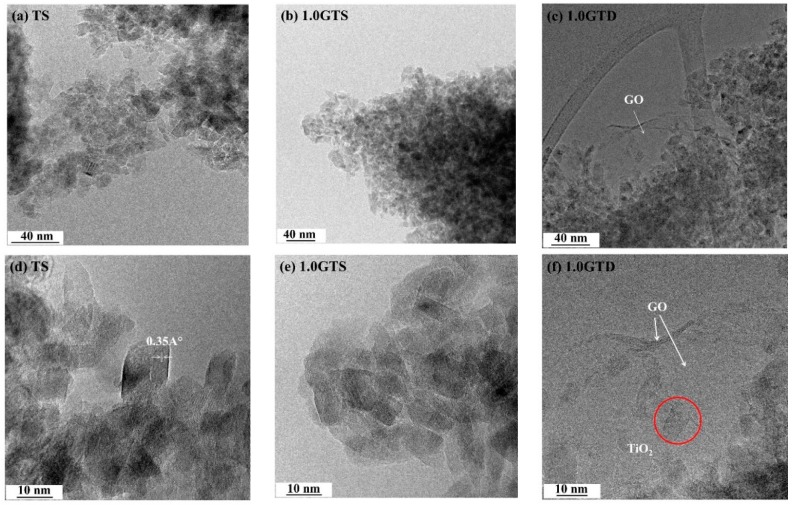
TEM images were taken at low (first row) and high magnification (second row) of the samples prepared under hydrothermal process: (**a**,**d**) the pure TiO_2_ prepared at 100 °C (TS); and GT composites of 1.0 wt % GO prepared at 100 °C: (**b**,**e**) statistic, 1.0GTS; and (**c**,**f**) dynamic, 1.0GTD.

The morphology of pure TiO_2_ and the GT composites was observed by the TEM analysis. The pure titania sample (TS) consisted of highly agglomerated TiO_2_ nanoparticles (Figure 2a) with polyhedral shape and crystal size of 10–1 2 nm (Figure 2b). The same morphology appeared in 1.0GTS (Figure 2b,e) and 1.0GTD (Figure 2c,f)). As expected, GO sheets were covered by aggregated TiO_2_ particles, as their functional groups acted as nucleation centers. Due to the low concentration, GO sheets were not stacked in nanoribbon structures and the composites consisted of TiO_2_ nanoparticles laying over crumpled GO monolayers, one of which is indicated by the white arrows in Figure 2c,f.

**Figure 3:**
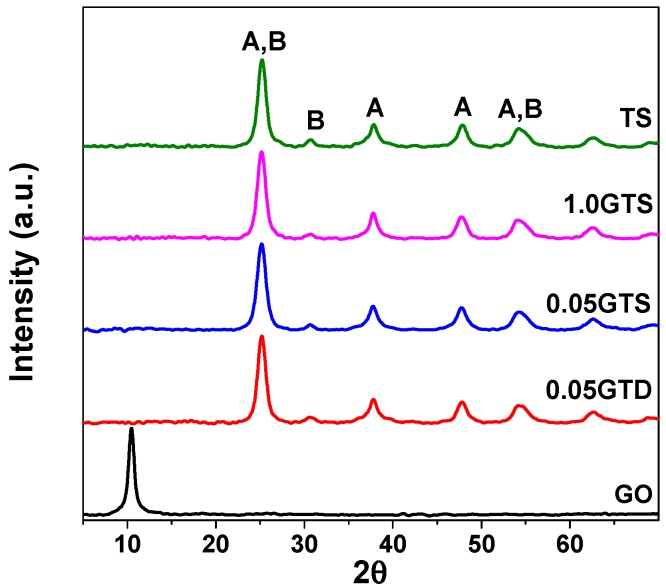
XRD patterns of starting GO, pure TiO_2_ (TS) and GT composite materials prepared at 100 °C by dynamic (0.05GTD) and statistic methods (0.05GTS and 1.0GTS). A and B represent the characteristic peaks of anatase and brookite phases, respectively.

The XRD results of the starting GO, pure TiO_2_ (TS) and GT composites synthesized in static conditions (0.05GTS and 1.0GTS) and under stirring (0.05GTD) are shown in Figure 3. GO presented the typical peak of graphene oxide at 2θ = 10.46° [31]. However, this peak disappeared in the GT composites due to the low concentration and exfoliation of GO [25,32]. All composite materials showed the characteristic peaks of anatase (A) and trace of brookite (B) at the 2θ angle between 25° and 70°. The anatase phase fraction was calculated to be around 75% for pure TiO_2_ and 80% for GT composites, irrespective of the synthesis method: dynamic or statistic. In addition, the GO did not affect the anatase fraction, in agreement with our previous findings [10].

**Figure 4:**
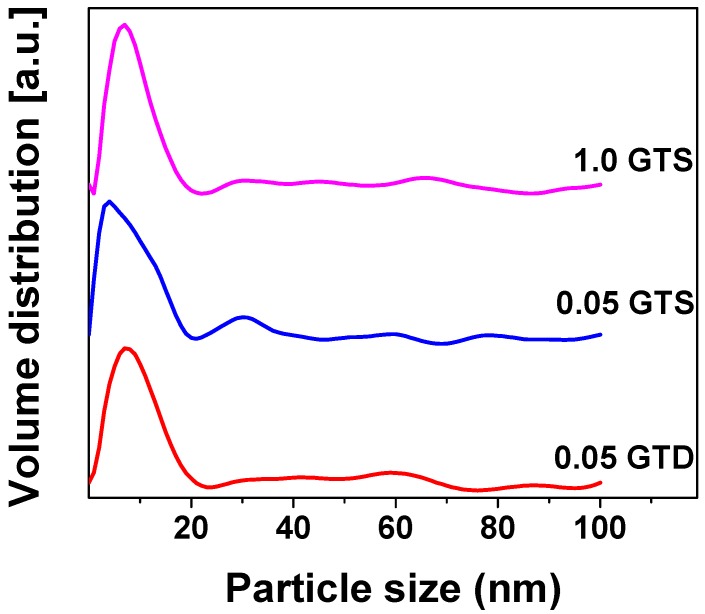
TiO_2_ particle size distribution of GT composites by SAXS analysis.

The particle size of TiO_2_ as measured by SAXS analysis is shown in Figure 4. No detectable change in crystalline size was observed by changing the GO loading from 0.0 to 1.0 wt %. All samples were found to consist of crystallites with average size of 8–9 nm.

**Figure 5:**
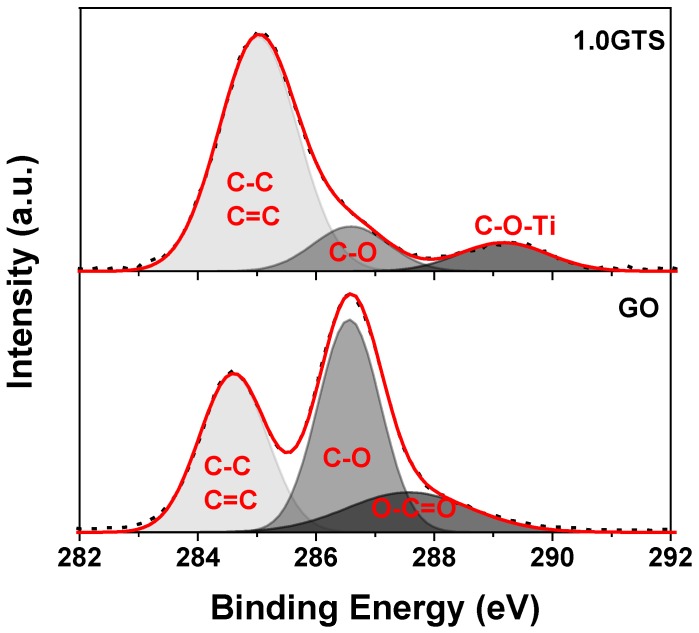
XPS of C*1_S_* of GO and 1.0GTS.

Figure 5 shows the C*1s* XPS spectra of the starting GO and of the GT composites. After deconvolution, the characteristic peak of the non-oxidized graphitic *sp*^2^ carbon atoms appeared at binding energy around 284.6 eV and two peaks of oxidized carbon point around 286.5 and 287.7 eV (C-O and O-C=O, respectively) in pure GO [33]. The presence of these types of oxidized carbon atoms is consistent with the structure of GO and is functional to the synthesis of the composites. Indeed, carboxylic acid groups can interact with Ti^4+^ ions and titania clusters during synthesis. As a consequence, in the XPS C1s spectrum of 1.0GTS, the biding energy of the carboxyl carbon (O-C=O) is shifted to the higher energy at ~289.5 eV, due to the formation of Ti-O-C bonding [13,34]. Moreover, the area ratios of the oxidized carbon atoms (A_C-O_ and A_O-C=O_) over reduced carbon (A_C-C_) show that GO was partially reduced during the synthesis of the GT composites. Indeed, the A_C-O_/A_C-C_ and A_O-C=O_/A_C-C_ ratios are, respectively, 1.3 and 0.40 for pure GO, dropping to 0.65 and 0.24 for the 1.0GTS sample. A similar result was observed in the sample prepared with 1.0 wt % loading in a stirred reactor, for which the following peak ratios were measured: A_C-O_/A_C-C_ = 0.54 and A_O-C=O_/A_C-C_ = 0.27. Unfortunately, it was not possible to analyze samples with a GO loading lower than 1.0 wt % because, in this sample, the GO signal was weaker than the background arising from the ubiquitous carbon contamination [35]. Nevertheless, the data of the samples prepared with 1.0 wt % GO loading show that about 50% of oxygen functional groups were removed during synthesis. We already observed this phenomenon [10], which can be explained by considering that functional groups on the GO plane simultaneously act as nucleation center and oxygen source for the growth of TiO_2_ nanoparticles.

### 3.2. Photocatalytic Activity 

**Figure 6:**
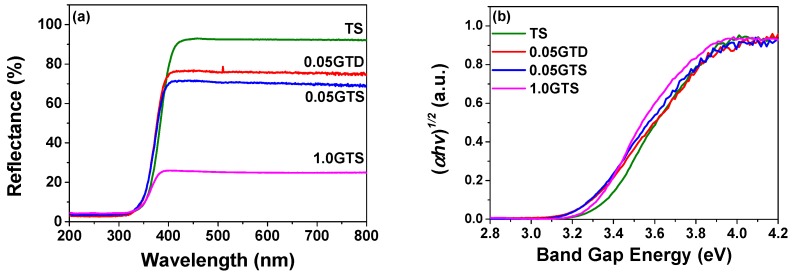
(**a**) UV-visible diffuse reflectance spectra within 200–800 nm; and (**b**) modified Kubelka–Munk (${\left(\alpha hv\right)}^{\frac{1}{2}}$) and band gap energy of pure TiO_2_ and the GT composites.

The UV-Vis reflection spectra of pure TiO_2_ and GT composites are depicted in Figure 6a. At wavelengths above 400 nm, pure TiO_2_ presents the lowest absorption, while the light absorption of the composites increases with the GO loading. In addition, the absorption edge of all nanocomposites shows a small red-shift compared with the pure TiO_2_ powder, as shown in Figure 6b. Indeed, from the linear extrapolation method [27,36,37,38], band gap energy was estimated to be 3.3 eV for TS and 3.2 for the GT nanocomposites. The narrowing of band gap energy can be ascribed to the electron transfer from TiO_2_ to GO via Ti-O-C bonding [17,37]. The small reduction of the apparent band gap energy in GT composites, corresponding to a bathochromic shift from 376 nm to 388 nm, can be ascribed to the superposition of absorption spectra of two different materials or to the generation of a limited number of localized intraband gap states [20]. In our case, no significant difference in band gap energy was observed for the GT samples with a GO loading between 0.05 and 1.0 wt %, suggesting that the GO added above 0.05 wt %, did not create new interface with the TiO_2_ nanoparticles, but rather formed stacked structures together with other GO sheets.

**Figure 7:**
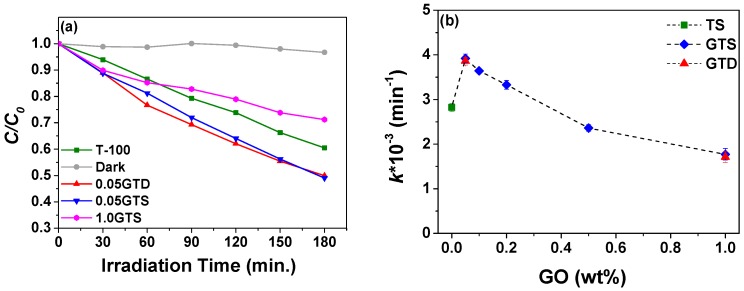
(**a**) Phenol degradation under sun simulator of TiO_2_ and GT composites via dynamic and statistic synthesis; and (**b**) k constant of difference amount of GO to TiO_2_. Vertical bars indicate error in the fitting of the data in Figure 1a with a pseudo-first order kinetic function. Standard deviation of *k* values measured on three kinetic tests were smaller than error bars.

Since the GO interaction with TiO_2_ appeared to have a beneficial effect on the band gap of the semiconductor, but the high absorption of GO could also limit the photodegradation activity, we studied the degradation of 10 ppm phenol with different GO loading in GT composites under sun simulator, as shown in Figure 7. Pure TiO_2_ prepared at 100 °C under static conditions was used as reference, while the composites prepared at 0.05 and 1.0 wt % of GO in a stirred reactor (0.05 and 1.0GTD) were used for comparing the different methods of synthesis. Control tests performed in the dark did not show significant phenol abatement, meaning that GT composites and phenol did not react in the absence of light. The plot of the normalized concentration ($\frac{C}{C_{0}}$) versus irradiation time in Figure 7a illustrates that the 0.05GTS and 0.05GTD achieved more than 50% abatement of phenol after 180 min, while only 40% of phenol degradation was reached with the TS reference in the same period. On the contrary, the photocatalytic efficiency of both GO composites with 1.0 wt % GO loading was the lowest: only around 25% of phenol was degraded after 180 min of exposure to the simulated sunlight.

The kinetic constant (k) of the photocatalytic degradation rate was calculated according to a pseudo-first-order reaction mechanism. The k values determined for composites with different GO loading and for the pure TiO_2_ reference are shown in Figure 7b. The samples with a 0.05 wt % GO loading show the highest photocatalytic rate, i.e., approximately 3.92 ± 0.1 × 10^−3^ min^−1^ and 3.86 ± 0.1 × 10^−3^ min^−1^ for the samples prepared in the static and stirred reactor, respectively. Such values are comparable to those reported in the literature for samples tested under simulated solar light [19,20]. This result can be explained by considering that the presence of GO on the surface of the photocatalyst can enhance the light absorption by lowering the band gap energy and it can hinder electron–hole recombination [12]. However, k declines with increasing the amount of GO and samples with GO loading ≥0.5 wt % have lower activity than pure TiO_2_. We expected this trend, since GO can shield the TiO_2_ particles from the light [17,18], as supported from the UV-visible absorption data discussed above. The data in Figure 7b also indicate that the degradation rates of the composite materials synthesized under stirring (GTD) were not significantly different from those measured for the corresponding samples prepared under static conditions.

## 4. Conclusions

GT composites were synthesized by the sol-gel method with two different reactor configurations: (a) stirring mixture in Pyrex beaker; and (b) a static condition in autoclave vessel. GO loading was varied from 0 to 1.0 wt %. All materials were prepared at 100 °C and consisted of TiO_2_ particles with polyhedral shape and 8–9 nm crystallite size, agglomerated on GO monolayers. XRD analysis revealed that all samples were mixtures of anatase and brookite phases, with a prevalence of anatase (around 75–80%). GT composites prepared with different methods but same GO loading showed similar morphology. XPS analysis suggested the formation of Ti-O-C interface bonding, because the peak corresponding to the oxidized carboxylic groups in the GT composites shifted towards higher binding energy, compared to the starting GO. Moreover, all GT composites showed a bathochromic shift of their absorption edge (from 376 nm to 388 nm), thus showing higher ability to exploit solar light (lower ban gap). Nevertheless, the photodegradation efficiency of the GT composites decreased with the GO loading. There are two possible reasons for that [38]: Firstly, GO acted as a light absorber, thus competing with the TiO_2_ photocatalytic centers [39,40]. Secondly, at high loading, GO acted as a charge carrier recombination center, thus facilitating the electron–hole pare recombination [18,41]. Therefore, our study stressed the importance TiO_2_-GO interface for substrates such as phenol, which shows no significant adsorption on GO, a common feature of most water micropollutants. Moreover, our study showed that the different methods, i.e. dynamic and static mixing in the reactor, did not influence the morphology and chemistry of GT nanocomposites. The photocatalytic activity was ruled mainly by the GO loading.

In this work, we prepared GT composites economically, with low-energy and low-chemical consumption, nearly neutral pH, and environmentally friendly syntheses. Our materials can be produced with a constant structure, even by using different types of reactor for the synthesis. The synergy between GO and TiO_2_ appeared to depend on interface. Therefore, the materials with the highest phenol photodegradation activities were those with 0.05 wt % GO loading. On the contrary, materials with GO loading higher than 0.5 wt % had lower activity than the pure TiO_2_ reference.

## References

[1] WWAP (United Nations World Water Assessment Programme) (2017). The United Nations World Water Development Report 2017. Wastewater: The Untapped Resource.

[2] Anku W.W., Mamo M.A., Govender P.P. (2017). Phenolic Compounds—Natural Sources, Importance and Applications.

[3] Rafiee E., Noori E., Zinatizadeh A.A., Zanganeh H. (2016). Photocatalytic degradation of phenol using a new developed TiO_2_/Graphene/Heteropoly acid nanocomposite: Synthesis, characterization and process optimization. RSC Adv..

[4] Villegas L.G.C., Mashhadi N., Chen M., Mukherjee D., Taylor K.E., Biswas N. (2016). A short review of techniques for phenol removal from wastewater. Curr. Pollut. Rep..

[5] Adamu H., Dubey P., Anderson J.A. (2016). Probing the role of thermally reduced graphene oxide in enhancing performance of TiO_2_ in photocatalytic phenol removal from aqueous environments. Chem. Eng. J..

[6] Faraldos M., Bahamonde A. (2017). Environmental applications of titania-graphene photocatalysts. Catal. Today.

[7] Jiang Y., Wang W.-N., Liu D., Nie Y., Li W., Wu J., Zhang F., Biswas P., Fortner J.D. (2015). Engineered crumpled graphene oxide nanocomposite membrane assemblies for advanced water treatment processes. Environ. Sci. Technol..

[8] Ola O., Maroto-Valer M.M. (2015). Review of material design and reactor engineering on TiO_2_ photocatalysis for CO_2_ reduction. J. Photochem. Photobiol. C Photochem. Rev..

[9] Giovannetti R., Rommozzi E., Zannotti M., D’Amato C.A. (2017). Recent advances in graphene based TiO_2_ nanocomposites (GTiO_2_Ns) for photocatalytic degradation of synthetic dyes. Catalysts.

[10] Naknikham U., Boffa V., Magnacca G., Qiao A., Jensen L.R., Yue Y. (2017). Mutual-stabilization in chemically bonded graphene oxide–TiO_2_ heterostructures synthesized by a sol–gel approach. RSC Adv..

[11] Li X., Yu J., Wageh S., Al-Ghamdi A.A., Xie J. (2016). Graphene in photocatalysis: A review. Small.

[12] Atout H., Álvarez M.G., Chebli D., Bouguettoucha A., Tichit D., Llorca J., Medina F. (2017). Enhanced photocatalytic degradation of methylene blue: Preparation of TiO_2_/Reduced graphene oxide nanocomposites by direct sol-gel and hydrothermal methods. Mater. Res. Bull..

[13] Jing J., Zhang Y., Li W., Yu W.W. (2014). Visible light driven photodegradation of quinoline over TiO_2_/Graphene oxide nanocomposites. J. Catal..

[14] Min Y., Zhang K., Zhao W., Zheng F., Chen Y., Zhang Y. (2012). Enhanced chemical interaction between TiO_2_ and graphene oxide for photocatalytic decolorization of methylene blue. Chem. Eng. J..

[15] Huang Q., Tian S., Zeng D., Wang X., Song W., Li Y., Xiao W., Xie C. (2013). Enhanced photocatalytic activity of chemically bonded TiO_2_/Graphene composites based on the effective interfacial charge transfer through the C–Ti Bond. ACS Catal..

[16] Liu Y. (2014). Hydrothermal synthesis of TiO_2_–RGO composites and their improved photocatalytic activity in visible light. RSC Adv..

[17] Nawaz M., Miran W., Jang J., Lee D.S. (2017). One-step hydrothermal synthesis of porous 3D reduced graphene Oxide/TiO_2_ aerogel for carbamazepine photodegradation in aqueous solution. Appl. Catal. B Environ..

[18] Pan D., Jiao J., Li Z., Guo Y., Feng C., Liu Y., Wang L., Wu M. (2015). Efficient separation of electron–hole pairs in graphene quantum dots by TiO_2_ heterojunctions for dye degradation. ACS Sustain. Chem. Eng..

[19] Wang P., Wang J., Wang X., Yu H., Yu J., Lei M., Wang Y. (2013). One-Step synthesis of easy-recycling TiO_2_-RGO nanocomposite photocatalysts with enhanced photocatalytic activity. Appl. Catal. B Environ..

[20] Minella M., Sordello F., Minero C. (2017). Photocatalytic process in TiO_2_/Graphene hybrid materials. Evidence of charge separation by electron transfer from reduced graphene oxide to TiO_2_. Catal. Today.

[21] El-Sheikh S.M., Khedr T.M., Hakki A., Ismail A.A., Badawy W.A., Bahnemann D.W. (2017). Visible light activated carbon and nitrogen Co-Doped mesoporous TiO_2_ as efficient photocatalyst for degradation of ibuprofen. Sep. Purif. Technol..

[22] Choina J., Kosslick H., Fischer C., Flechsig G.-U., Frunza L., Schulz A. (2013). Photocatalytic decomposition of pharmaceutical ibuprofen pollutions in water over titania catalyst. Appl. Catal. B Environ..

[23] Wang T., Xu Z., Wu L., Li B., Chen M., Xue S., Zhu Y., Cai J. (2017). Enhanced photocatalytic activity for degrading phenol in seawater by TiO_2_-based catalysts under weak light irradiation. RSC Adv..

[24] Zhang H., Banfield J.F. (2000). Understanding polymorphic phase transformation behavior during growth of nanocrystalline aggregates:  Insights from TiO_2_. J. Phys. Chem. B.

[25] Wang W.-N., Jiang Y., Biswas P. (2012). Evaporation-induced crumpling of graphene oxide nanosheets in aerosolized droplets: Confinement force relationship. J. Phys. Chem. Lett..

[26] Ünlü H., Horing N.J.M., Dabowski J. (2015). Low-Dimensional and Nanostructured Materials and Devices: Properties, Synthesis, Characterization, Modelling and Applications.

[27] Khannam M., Sharma S., Dolui S., Dolui S.K. (2016). A graphene oxide incorporated TiO_2_ photoanode for high efficiency quasi solid state dye sensitized solar cells based on a poly-vinyl alcohol gel electrolyte. RSC Adv..

[28] López R., Gómez R. (2012). Band-Gap energy estimation from diffuse reflectance measurements on sol–gel and commercial TiO_2_: A comparative study. J. Sol-Gel Sci. Technol..

[29] Malekshoar G., Pal K., He Q., Yu A., Ray A.K. (2014). Enhanced solar photocatalytic degradation of phenol with coupled graphene-based titanium dioxide and zinc oxide. Ind. Eng. Chem. Res..

[30] Zhang Y., Zhou Z., Chen T., Wang H., Lu W. (2014). Graphene TiO_2_ nanocomposites with high photocatalytic activity for the degradation of sodium pentachlorophenol. J. Environ. Sci..

[31] Azarang M., Shuhaimi A., Sookhakian M. (2015). Crystalline quality assessment, photocurrent response and optical properties of reduced graphene oxide uniformly decorated zinc oxide nanoparticles based on the graphene oxide concentration. RSC Adv..

[32] Zhang X., Kumar P.S., Aravindan V., Liu H.H., Sundaramurthy J., Mhaisalkar S.G., Duong H.M., Ramakrishna S., Madhavi S. (2012). Electrospun TiO_2_–graphene composite nanofibers as a highly durable insertion anode for lithium ion batteries. J. Phys. Chem. C.

[33] Zhang J., Xiong Z., Zhao X.S. (2011). Graphene–metal–oxide composites for the degradation of dyes under visible light irradiation. J. Mater. Chem..

[34] Peng D., Qin W., Wu X., Wu J., Pan Y. (2015). Improvement of the resistance performance of carbon/cyanate ester composites during vacuum electron radiation by reduced graphene oxide modified TiO_2_. RSC Adv..

[35] Chen P., Wang L., Wang P., Kostka A., Wark M., Muhler M., Beranek R. (2015). CNT-TiO_2_−δ composites for improved co-catalyst dispersion and stabilized photocatalytic hydrogen production. Catalysts.

[36] Štengl V., Bakardjieva S., Grygar T.M., Bludská J., Kormunda M. (2013). TiO_2_-graphene oxide nanocomposite as advanced photocatalytic materials. Chem. Cent. J..

[37] Pei F., Liu Y., Zhang L., Wang S., Xu S., Cao S. (2013). TiO_2_ nanocomposite with reduced graphene oxide through facile blending and its photocatalytic behavior for hydrogen evolution. Mater. Res. Bull..

[38] Wang D., Li X., Chen J., Tao X. (2012). Enhanced photoelectrocatalytic activity of reduced graphene oxide/TiO_2_ composite films for dye degradation. Chem. Eng. J..

[39] Xiang Q., Yu J., Jaroniec M. (2011). Preparation and enhanced visible-light photocatalytic H_2_-production activity of graphene/C_3_N_4_ composites. J. Phys. Chem. C.

[40] Zhu G., Xu T., Lv T., Pan L., Zhao Q., Sun Z. (2011). Graphene-incorporated nanocrystalline TiO_2_ films for CdS quantum dot-sensitized solar cells. J. Electroanal. Chem..

[41] Ai Z., Ho W., Lee S. (2011). Efficient visible light photocatalytic removal of NO with BiOBr-graphene nanocomposites. J. Phys. Chem. C.

